# Digital Rectal Examination Standardization for Inexperienced Hands: Teaching Medical Students

**DOI:** 10.1155/2013/797096

**Published:** 2013-09-19

**Authors:** Leonardo Oliveira Reis, Antonio Felipe Leite Simão, Jamal Baracat, Fernandes Denardi, Antonio Gugliotta

**Affiliations:** ^1^Faculty of Medical Sciences, University of Campinas, Unicamp, Rua Tessália Vieira de Camargo 126, Cidade Universitária “Zeferino Vaz”, 13083-887 Campinas, SP, Brazil; ^2^Faculty of Medicine, Center for Life Sciences, Pontifical Catholic University of Campinas, PUC-Campinas, 13060-904 Campinas, SP, Brazil

## Abstract

*Objectives.* To standardize digital rectal examination (DRE) and set how it correlates with the comprehensive evaluation of lower urinary tract symptoms (LUTS). *Methods.* After scaled standardization of DRE based on fingertips graphical schema: 10 cubic centimeters—cc for each fingertip prostate surface area on DRE, four randomly selected senior medical students examined 48 male patients presenting with LUTS in an outpatient clinical setting, totaling 12 DRE each. Standardized DRE, international prostate symptom score (IPSS), serum PSA, transabdominal ultrasound (US), urodynamic evaluation, and postvoid residue were compared. *Results.* The mean and median PVs were US—45 and 34.7 cc (5.5 to 155) and DRE—39 and 37.5 cc (15 to 80). Comparing DRE and US by simple linear regression: US PV = 11.93 + 0.85 × (DRE PV); *P* = 0.0009. Among patients classified as nonobstructed, inconclusive, and obstructed, the US PVs were 29.8, 43.2, and 53.6 cc (*P* = 0.033), and DRE PVs were 20, 35, and 60 cc (*P* = 0.026), respectively. *Conclusion.* This is the first attempt to DRE standardization focusing on teaching-learning process, establishing a linear correlation of DRE and US PVs with only 12 examinations by inexperienced hands, satisfactorily validated in an outpatient clinical setting.

## 1. Introduction

 After anamnesis, clinical evaluation with physical examination is fundamental to proceed with patient investigation, determining the necessary complementary exams and even defining treatments. 

 Classically, the initial approach to men presenting with low urinary tract symptoms (LUTS) is accomplished by digital rectal examination (DRE), prostate specific antigen (PSA), international prostatic symptom score (IPSS), and postvoid residue by ultrasonography (US) [1]. 

 The DRE technique is a simple and well-established maneuver; however, this propaedeutic method and mainly its optimal quantification of prostate volume (PV) still remain empirical knowledge, with no scientific reasoning and standardization. PV has a direct correlation with natural history of prostate enlargement and subsequent risk of a poor outcome [1].

 In this scenario, standardized, simple, fast, low cost, and effective methods for teaching inexperienced physicians on DRE ability are desirable, considering the recognized importance of DRE in terms of valuable information to direct patient treatment and the fact that this aspect of clinical examination is frequently relegated to the specialist [2].

 At the expense of inefficient DRE, ultrasonographic parameters are the central method of assessing male LUTS [3], and it is not yet well established how the clinical examination of the prostate can contribute to the assessment of PV and how such data can be applied in the management of patients with LUTS in primary care by newly formed physicians and general practitioners [4].

 This preliminary study evaluates the impact of DRE standardization on inexperienced hands and sets how the designed method correlates with the comprehensive evaluation of men presenting with LUTS to assess the acquisition and validity of DRE skills, mainly PV assessment.

## 2. Methods

### 2.1. DRE Standardization

 During years of clinical experience, confronting prostate volume estimated by DRE and US, it was found by empirical observation that, although assessing only the posterior surface area of a three-dimensional structure, DRE correlates with overall prostate volume. 

 Based on the premise that posterior surface area has a high predictive value for overall prostate volume and is focused on the teaching-learning process of medical clinical practice and propaedeutic of physical examination, scaled standardization of clinical impression of PV by DRE was developed based on fingertips graphical schema.

**Figure 1:**
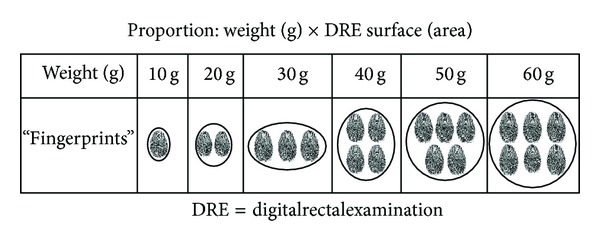
Scaled standardization of clinical impression of prostate weight (1 g = 1 cc) by DRE based on fingertips graphical schema.

 For each fingertip of prostate surface area (width and length of the posterior surface) on DRE, the examiner was guided to consider 10 cubic centimeters (cc) of prostate tissue (Figure 1).

### 2.2. Model Validation

 In accordance with institutional ethical guidelines, based on good clinical practice, four randomly selected senior medical students were exposed to a 10-minute lecture presentation on DRE practice, in which the scaled standardization of clinical impression of PV by DRE (Figure 1) was demonstrated in the simulated pelvic model with a prostate model relative to average dimensions of different prostate volume, but focusing on the two-dimensional posterior surface, which is accessible in DRE. 

 Thereafter, by informed consent, they examined 48 subsequent male patients presenting with LUTS potentially associated with benign prostatic hyperplasia (BPH) in an outpatient clinical setting, totaling 12 DRE each. All DRE were performed in the standing-up position. To determine the number of DRE per student in the study design, the fact that most of them perform less than ten examinations during graduation was considered [5].

 International prostate symptom score (IPSS), physical examination (standardized DRE), serum PSA (obtained before DRE), transabdominal ultrasound (US), urodynamic evaluation (Dynapack, Dynamed, 2004), and postvoiding residue were compared. 

 All ultrasound examinations were performed with bladder volume of 100–200 mL, by single experienced radiologist unaware of the DRE results, using Toshiba 6000 model Power Vision in a sagittal plane with frequency transducers 3–6 MHz, and prostate volume was calculated by the prostate ellipsoid formula (0.52 × width × length × height). Considering the prostate gravity of approximately 1.0, we compared volume estimates by equating 1 cc to 1 g.

 According to the bladder outlet obstruction index (BOOI) [6], patients were classified into nonobstructed, inconclusive, and obstructed: BOOI < 20, BOOI = 20–40, and BOOI > 40, respectively.

 As statistical methodology, a descriptive analysis was performed using measurements of position and dispersion for continuous variables as measure of linear association between PV estimations by DRE and US. 

 A simple linear regression analysis and Bland-Altman plots were used to verify the degree of agreement between the measurements by DRE and US. The Kruskal-Wallis test was used to compare continuous measures among three groups—obstructed, nonobstructed, and inconclusive—regarding PV by DRE and by US classifications. The level of significance was set at 5%.

## 3. Results

The mean age of the examined patients was 64.9 years (56–73); PSA values ranged from 1.2 to 5.4 with a mean of 4.3, mean IPSS of 13, ranging from 6 to 20, and mean postvoiding residue of 70 mL, ranging from 0 to 250 mL (Table 1).

**Figure 2:**
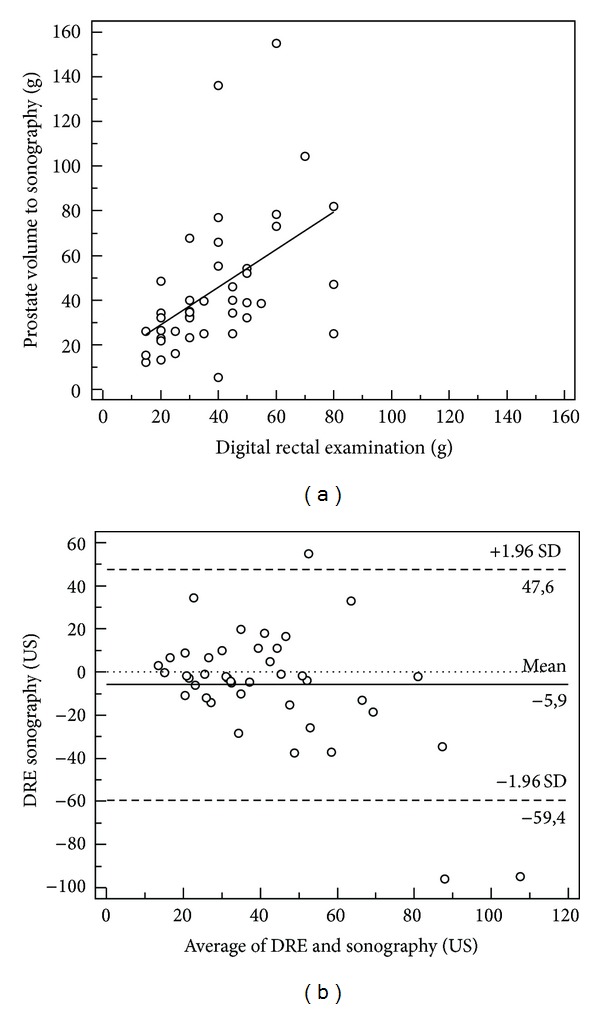
Correlation between prostate volume (PV) estimations by digital rectal examinations (DRE) and ultrasonography (US) and Bland and Altman plot, (1 g = 1 cc).

 The mean and median prostate volumes were, respectively, 45 and 34.7 cc (5.5–155) in ultrasonography evaluation (US) and 39 and 37.5 cc (15–80) in DRE, with a reliable correlation between PV estimations by DRE and US at Bland and Altman plot (Figure 2).

 The positive predictive value to identify prostates above 30 cc, a clinically significant indication of 5-alpha reductase inhibitors, was 92.3% (24 of 26 cases).

 Applying simple linear regression to compare the two methods (DRE and US), depending on the model: US  PV = *a* + *b* × (DRE  PV), we have

(1) $$\begin{array}{c} \text{US}\;\text{PV}=11.93+0.85\times \left(\text{DRE}\;\text{PV}\right);\;P=0.0009. \end{array}$$

Among patients classified as nonobstructed, inconclusive, and obstructed, there was a significant correlation between PV estimated by DRE (*P* = 0.026) and US (*P* = 0.033) with BOOI (Table 2).

## 4. Discussion

### 4.1. Teaching-Learning DRE

 DRE maneuver is already well established but persists in a field of empirical knowledge and requires standardization that will permit knowledge dissemination in a unified and understandable method [7, 8].

 While the teaching mannequin remains the preferred instrument of teaching DRE, mannequin and even virtual reality-based simulations have fidelity limits; supervised patient examination is still perceived as the gold standard teaching experience; however, only limited knowledge exists regarding the technique of teaching and assessing DRE [7–9]. Also, cost constraints limit the availability of virtual reality and rectal teaching associates that may have also cultural restrictions [9]. 

 At the same time, currently we are offering less basic office evaluation, tending to propose complementary exams before examining the prostate, culminating certainly in cost increments with no warranted benefits [3]. Despite the use of new technology, clinical examination will always play a major role in the diagnosis of clinical problems. DRE needs standardization and validation for using abroad, and it is important for clinicians to know how closely one can estimate prostate size, as determined by US, using DRE [4].

### 4.2. Clinical Impact of DRE

 DRE of the prostate gland is an important diagnostic tool in the context of both benign and malignant diseases. With the growing aging population, especially in developing countries like Brazil, the significant increase of LUTS and also prostate cancer on male population [10] is indisputable.

Prostate size is a prognostic factor in deciding which surgical techniques and/or medical treatments may be the most appropriate for individual patients with LUTS [1]. Another important use of prostate volume is for the PSA density (PSAD) calculation, which is defined as total serum PSA divided by prostate gland weight. PSAD is an important tool for prostate cancer risk and staging [10]. 

### 4.3. Confronting Results with Literature

 Presented results showed a reasonable correlation of prostate volumes measured by transabdominal US and standardized DRE for inexperienced hands that has also correlated with comprehensive complete prostate workup. Still significantly, but to a lesser extent, prostatic volume obtained by ultrasound and DRE also related to the BOOI rank.

 Considering that transrectal methods can produce great discomfort to the patient, we have used abdominal ultrasound, a method equivalent to rectal ultrasound for measuring the prostate when bladder volume is over 100 mL [11, 12].

 Loeb et al. showed that, when estimated by multiple examiners with no previous standardization of the examination technique, DRE correlated poorly, while transrectal US estimated better the surgical specimen weight. Considering that US is performed in a more systematized and standardized way than DRE, incorporating DRE standardization to the clinical practice may change their conclusions in the future [13].

 Also supporting the need for standardization, Cheng et al. found that the trained urologist (over 5-year experience) was more accurate in estimating prostatic volume with DRE than the urology junior trainee (two months working in urology), envisaging a long learning curve and a wide interobserver variation, which may be potentially improved by optimizing the teaching-learning process by DRE standardization. At the same time, a tendency for accuracy improvement after performing more examinations is also expected after introducing DRE standardization [14], and future studies are warranted focusing particularly on the learning curve that is beyond the present study targets. 

 Concerning adequacy, the proposed bidimensional model is very satisfactory, since DRE accesses the prostate posterior surface area, which is bidimensional. Previous proposed models are three-dimensional relief models that are far from the DRE bidimensional clinical impression [7–9, 15].

 Standardizing DRE for a more accurate clinical impression of prostate volume in the physical examination will impact the entire medical community [6, 16]. Bosch et al. have shown that prostate volume estimation between 30 and 50 cc on DRE may be an acceptable method for monitoring in case of not available ultrasonography, given the good accuracy of the method in this range [4]. Recently, Ahmad et al. demonstrated that ultrasound would be required for volumes less than 30 cc or above 80 cc, while DRE has positive predictive value of 94% to identify prostates above 30 cc, a clinically significant indication of 5-alpha reductase inhibitors [17]. In our experience with DRE standardization, even for inexperienced hands, 92% patients (24 of 26 cases) were accurately estimated on DRE for clinical relevant prostate volumes (>30 cc), standing at a very little distance from ultrasonography findings when excluding outliers in ultrasound. 

 Kijvikai, in a systematic review, found that prostate volume by digital rectal examination, identifying large-volume prostates, is impactful to the natural history of benign prostatic hyperplasia, whereas prostate-specific antigen would be additional tool for predicting disease progression and guiding therapeutic options, being the prostate ultrasonography reserved for guiding eventual biopsy or surgical treatment [18]. 

 Thus, only clinical approaches would be enough to guide the therapeutic management of many patients, without the need for additional ultrasound, since DRE has good accuracy for medium-sized prostates [7–9, 15].

 Strengths of the current study include complete prostatic workup, prospective design, and an easy and intuitive standardization. Limitations include the fact that the examiners are only able to estimate the prostate size in increments of 10 cc and the relatively small number of examinations; however, given that DRE is assessing only the posterior surface area of a three-dimensional structure, smaller increments would be imprecise. Also, the study design was focused on newly formed physicians who, when well motivated, perform about 5–10 DRE only in their training program [5]. 

 This pilot study demonstrates the proof of principle in the setting of preliminary DRE learning. Future studies must be designed to contemplate different medical specialties with different expositions to DRE. The next step, a longer trial investigating how DRE accuracy evolves after preliminary learning, is currently underway, including different practices, denoting different exposures to DRE—higher to lower—as follows: urology, proctology, general surgery, emergency, and internal medicine. 

## 5. Conclusion

 This is the first attempt for digital rectal examination standardization satisfactorily validated in an outpatient clinical setting, focusing on the teaching-learning process. 

 Although DRE only provides a rough estimate of prostatic volume, when standardized, it is feasibly sufficient to classify patients and guide therapeutic options even in inexperienced hands.

## Figures and Tables

**Table 1:** Patients' clinical characteristics (1 cc = 1 g).

Clinical and demographic characteristics	Values—mean (range)
Age	64.9 (56 to 73) years
Caucasian Latin American	100%
PSA	4.3 (1.2 to 5.4) ng/mL
IPSS	13 (6 to 20)
Prostate volume (US)	45 (5.5 to 155) cc
Prostate volume (DRE)	39 (15 to 80) cc
Postvoiding residue	70 (0 to 250) mL

**Table 2:** BOOI ranking of PV US, PV DRE, PSA, IPSS, and PVR means.

Kruskal-Wallis test					PV-US (cc) *P* = 0.033		PV-DRE (cc) *P* = 0.026	
BOOI	*N*	PSA (ng/dL)	IPSS	PVR (mL)	Mean (cc)	SD	Mean (cc)	SD
Nonobstructed (<20)	12	1.5	6	15	29.8	19.4	20	10
Inconclusive (20–40)	14	2.3	8	30	43.2	33.1	35	15
Obstructed (>40)	22	4.5	17	120	53.6	32.9	60	15

BOOI: bladder outlet obstruction index.

PV: prostate volume.

US: transabdominal ultrasound.

DRE: standardized digital rectal examination.

SD: standard deviation.

IPSS: international prostatic symptom score.

PVR: postvoiding residue.

## References

[1] Kirby R (2011). Improving lower urinary tract symptoms in BPH. *Practitioner*.

[2] Naccarato AMEP, Reis LO, Matheus WE, Ferreira U, Denardi F (2011). Barriers to prostate cancer screening: psychological aspects and descriptive variables—is there a correlation?. *Aging Male*.

[3] Lim CH, Quinlan DM (2007). Are doctors examining prostates in university hospital?. *Urology*.

[4] Bosch JLHR, Bohnen AM, Groeneveld FPMJ (2004). Validity of digital rectal examination and serum prostate specific antigen in the estimation of prostate volume in community-based men aged 50 to 78 years: the Krimpen Study. *European Urology*.

[5] Fitzgerald D, Connolly SS, Kerin MJ (2007). Digital rectal examination: national survey of undergraduate medical training in Ireland. *Postgraduate Medical Journal*.

[6] Reis LO, Barreiro GC, Prudente A, Silva CM, Bassani JWM, D’Ancona CAL (2009). A novel intraurethral device diagnostic index to classify bladder outlet obstruction in men with lower urinary tract symptoms. *Advances in Urology*.

[7] Low-Beer N, Kinnison T, Baillie S, Bello F, Kneebone R, Higham J (2011). Hidden practice revealed: using task analysis and novel simulator design to evaluate the teaching of digital rectal examination. *American Journal of Surgery*.

[8] Balkissoon R, Blossfield K, Salud L, Ford D, Pugh C (2009). Lost in translation: unfolding medical students’ misconceptions of how to perform a clinical digital rectal examination. *American Journal of Surgery*.

[9] Popadiuk C, Pottle M, Curran V (2002). Teaching digital rectal examinations to medical students: an evaluation study of teaching methods. *Academic Medicine*.

[10] Reis LO, Zani EL, Alonso JC, Simões FA, Rejowski RF, Ferreira U (2011). Does the criterion for prostate biopsy indication impact its accuracy? A prospective population-based outpatient clinical setting study. *Actas Urologicas Espanolas*.

[11] Yuen JSP, Ngiap JTK, Cheng CWS, Foo KT (2002). Effects of bladder volume on transabdominal ultrasound measurements of intravesical prostatic protrusion and volume. *International Journal of Urology*.

[12] Ohnuki T, Kurokawa K, Katoh N (1987). Transrectal longitudinal ultrasonography of the prostate by electronic linear scanning. *Hinyokika Kiyo*.

[13] Loeb S, Han M, Roehl KA, Antenor JAV, Catalona WJ (2005). Accuracy of prostate weight estimation by digital rectal examination versus transrectal ultrasonography. *Journal of Urology*.

[14] Cheng WC, Ng FC, Chan KC, Cheung YH, Chan WL, Wong SW (2004). Interobserver variation of prostatic volume estimation with digital rectal examination by urological staffs with different experiences. *International Brazilian Journal of Urology*.

[15] Roehrborn CG, Sech S, Montoya J, Rhodes T, Girman CJ (2001). Interexaminer reliability and validity of a three-dimensional model to assess prostate volume by digital rectal examination. *Urology*.

[16] Reis LO, Barreiro GC, Baracat J, Prudente A, D’Ancona CA (2008). Intravesical protrusion of the prostate as a predictive method of bladder outlet obstruction. *International Brazilian Journal of Urology*.

[17] Ahmad S, Manecksha RP, Cullen IM (2011). Estimation of clinically significant prostate volumes by digital rectal examination: a comparative prospective study. *The Canadian Journal of Urology*.

[18] Kijvikai K (2009). Digital rectal examination, serum prostatic specific antigen or transrectal ultrasonography: the best tool to guide the treatment of men with benign prostatic hyperplasia. *Current Opinion in Urology*.

